# Removal of cadmium from a citrate-bearing solution by floatable microsized garlic peel†

**DOI:** 10.1039/c8ra03502d

**Published:** 2018-08-07

**Authors:** Jiangang Sun, Lipu Yin, Kai Huang, Xiaohui Li, Xianbin Ai, Ying Huang, Yanli Yin, Junyou Liu

**Affiliations:** School of Metallurgical and Ecological Engineering, University of Science and Technology Beijing Xueyuan Rd. 30, Haidian District 100083 Beijing City China khuang@metall.ustb.edu.cn +86-10-13552537538; Institute of Bioresource, Jiang Xi Academy of Sciences Changdong Rd. 7777 Nanchang City Jiang Xi Province China lixiaohui211121@163.com +86-15727656430; Beijing Keda Advanced Technology Company Xueyuan Rd. 30, Haidian District 100083 Beijing City China

## Abstract

Paddy field soil contaminated by cadmium may produce cadmium-containing corn due to the paddy's strong ability to accumulate cadmium. The *in situ* washing of soil with an organic acid is one of the suitable technical choices for the remediation of cadmium-contaminated soils. The limiting factor of this method lies in the recycling and reuse of the huge amount of washing effluent in an efficient and economical way. In present study, the simulated solutions were used to examine the adsorption efficiency of cadmium on a biosorbent which was synthesized by using garlic peel as the raw material. The biosorption behavior of cadmium on garlic peel was systematically studied in the presence of a citrate ligand. Presented here for the first time, garlic peel with buoyant properties was carefully collected and used for the preparation of the adsorbent, and verified to have a prominent advantage in efficiently separating from the solution after adsorption because of its floatability. Results show that the presence of citrate has a significant inhibition effect on the adsorption behavior of cadmium on the floating garlic peel, at the optimal pH of 4.0, which can be ascribed to the competitive affinity to the cadmium from the citrate ligand. SEM shows that floating garlic peel has a ruffled epidermis in the flat surface and porous microstructure in the transversal surface, making it durable enough and favorable for adsorption; and –COOH was determined by FTIR to be the main functional group contributing to the adsorption capability of garlic peel. Cadmium can be eluted off the garlic peel after adsorption, and the garlic peel can be then reused for the next cycle of adsorption with little decrease in adsorption capacity, even after ten adsorption/desorption cycles. The real leach liquor of cadmium-contaminated soil sample by 0.01 mol L^−1^ citric acid solution was used for testing, and it was found that after three adsorption uses, almost all the cadmium in the leach liquor had been recovered by the floating garlic peel. The above research results provided a possible route to recycle the soil washing solution by biosorption, giving a great perspective in the remediation of paddy field soil contaminated by cadmium.

## Abstract 2

Garlic peel was chosen as an effective adsorbent for a cadmium-contaminated soil remediation process.

## Graphical abstract

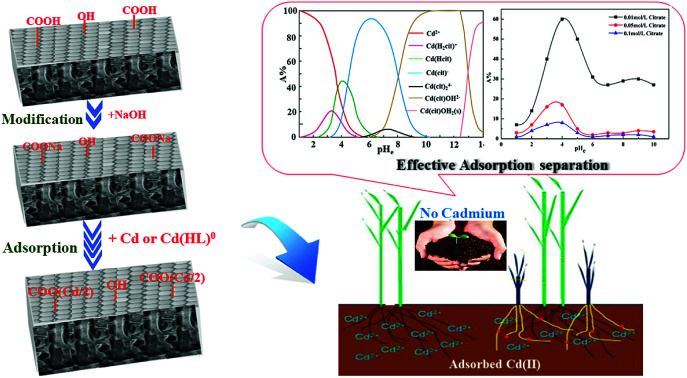

## 1. Introduction

Cadmium is a highly toxic heavy metal, often found accumulated in corn paddy fields in areas near smelting factories, battery plants or mineral mining districts where the cadmium-bearing effluents were frequently discharged in an uncontrolled manner.^1,2^ Another main source of cadmium to paddy fields is found in chemical fertilizers due to their mineral components.^3^ Related research has disclosed that rice crops have an especially strong capability to enrich cadmium in the corn from the cadmium contaminated paddy fields.^4,5^ One method is to plant a new type of paddy corn, which is developed by genetic engineering and has a low cadmium intake ability from the soils.^6–8^ The more practical method is to remediate the paddy field soils by some cost-effective technologies, such as soil replacement;^9,10^ immobilization by neutralization^11–13^ or by adsorption^14,15^ with various natural or industrial materials; electrokinetic migration processes;^16–18^ and chemical washing by special reagents.^19–22^

Of the above soil remediation techniques, soil replacement is simple, but it is difficult to imagine that enormous amount of soil can be dug, moved and safely treated in an economical way. Therefore, this method is suitable only for a small area of seriously polluted soil. Immobilization of the cadmium species by neutralization or by adsorption is currently the most frequently applied technique, in which CaO, zeolite, biochar and some natural mineral materials are used^11–15^ through the chemical precipitation and adsorption mechanism; the captured cadmium species may be released again into the soil, however, and its permanent safety assessment has seldom been studied and reported.^23^ The operational cost and effectiveness of the electrokinetic migration process may make it impractical in complex paddy field conditions.^16^*In situ* chemical washing of the soil is regarded as the best method because it has the advantages of easy operation on a large scale and it cleans the soil permanently. *In situ* chemical washing also presents some disadvantages that may limit its extensive application, such as a large volume of water required for washing, possible leakage into the groundwater aquifer, and lack of recycling techniques for the cadmium-bearing effluent produced in the washing process. In paddy fields, there are waterproof layers under the plow horizon that can prevent the potential percolation of the washing effluent from polluting the groundwater.^24,25^ Therefore, the key to this technique lies in whether the washing effluent can be effectively recycled and reused, the main challenge being how to remove the diluted cadmium from the huge volume of washing solution. For this washing solution, the biosorption has outstanding advantages for its treatment in an effective and economical way,^26–30^ but few reports about this effluent treatment technique have been found. If it works efficiently, *in situ* soil washing technique by chemical washing may find greater application in the remediation of cadmium-contaminated soil on large scale.

In the present study, we attempted to use garlic peel, a common agricultural waste, as the raw material to adsorb the cadmium from an artificial solution containing citric acid, which is frequently used as the leaching reagent for soil washing. To improve the solid/liquid separation and collection of garlic peel particles during the adsorption process, the modified garlic peel particles were firstly classified according to their floatability, and those that could float on the solution were collected for the adsorption test. To increase the adsorption interface of the biosorbents, intensive grinding, milling and sedimentation processes were used, and a fine garlic peel powder on a micrometer scale size was prepared. As prepared, the biomass powder has good floating behavior and a large adsorption surface, which is quite different from and superior to traditional biosorbents. The systematical study on the adsorption behavior for cadmium in the presence of citric acid was conducted, and the relative mechanism was also discussed to provide the fundamental principles for the paddy field soil remediation by washing and biosorption technologies.

## 2. Materials and methods

### 2.1 Materials

All the chemicals used in this study were analytical grade without further purification, and the solutions were prepared with deionized water and filtered to remove any possible particulate contaminants. A soil sample was taken from a neighbor area near a smelting factory in Zhuzhou city, which was examined to contain 13.2 mg kg^−1^ cadmium on the basis of dried weight by completely dissolved a certain dried sample in the aqua regia, and the leach liquor was collected by filtering.

### 2.2 Preparation of the adsorbent

The garlic waste was washed with tap water to remove particulate dirt, sand and clay and then washed with distilled water. The saponification method was used for the preparation of the adsorption particles, that is, 100 g of dry garlic waste was crushed and mixed with 1000 mL ethanol for 24 h to remove pigments, volatile organic compounds and some low-molecular-weight polysaccharides. The suspension was then filtered, and the solid cake was mixed with distilled water containing 5.5 g sodium hydroxide. After agitation for approximately 24 h, it was filtered and repeatedly washed with distilled water to a neutral pH. The wet, filtered cake was dried in an air convection oven at 60 °C for 12 h. To improve the adsorption efficiency, the modified garlic peel was intensively ground and milled to a controlled particle size of 50–100 μm by the sedimentation method, and the floating garlic peel was collected for use as the adsorbent samples in the present study. The final garlic adsorbent product was designated floatable saponified garlic peel (FSGP) and used for the adsorption tests in this work.

### 2.3 Batch-wise adsorption tests

Unless stated otherwise, typically in the batch-wise adsorption tests, 50 mg FSGP dry particles and 50 mL solution (initial conc. 10 mg L^−1^ Cd^2+^ with 0.01 mol L^−1^ citric acid), were mixed and intensively shaken at 293 K for 0.5–10 h to reach equilibrium in a thermostatic air bath incubator reciprocating at 200 rpm. The initial pH value of the cadmium ion solution was adjusted with 0.1 mol dm^−3^ HNO_3_ and 0.1 mol dm^−3^ NaOH solutions, and the pH values during the adsorption process were kept near constant due to the buffering effect of citric ligands, although its main role is as the leaching reagent. After adsorption, the solution samples were filtered, and the cadmium ion concentration in the filtrated liquor was measured by an inductive coupled plasma emission spectrometer (715-ES, Varian, United States). All the required experimental solutions were diluted with 0.1 mol dm^−3^ HNO_3_ to the concentration levels suitable for the analysis. All experiments were performed in duplicate, at a minimum, and mean values were presented in all the cases investigated. The amount of adsorption for metal ions was calculated according to the following equations.^32^

1 *A* = (*C*_i_ − *C*_e_)/*C*_i_ × 100

2 *Q* = (*C*_i_ − *C*_e_) × *V*/*W*

where *A* is the adsorption percentage of metal ions on the particles (%), *Q* denotes the amount of objective ion adsorbed per unit mass of the adsorbent (mol kg^−1^), *V* is the solution volume (dm^3^), *W* is the dry mass of the adsorbent (kg), and *C*_i_ and *C*_e_ represent the objective ion concentrations in the solution before and after adsorption (mol dm^−3^), respectively.

### 2.4 Characterization

The morphology and structure of the prepared garlic peel samples were investigated by using field emission scanning electron microscopy (SEM, Zeiss Supra55, Germany) with an accelerating voltage of 20 kV and energy dispersive spectrometry (EDS). Fourier transform infrared (FTIR) spectra were obtained by using a Nicolet IS10 spectrometer (Thermo Fisher, USA) at 4 cm^−1^ resolution. XPS analysis was performed on an AXIS ULTRA^DLD^ system, using monochromatic Al Kα radiation (1486.6 eV).

## 3. Results and discussion

### 3.1 SEM/EDS

**Fig. 1:**
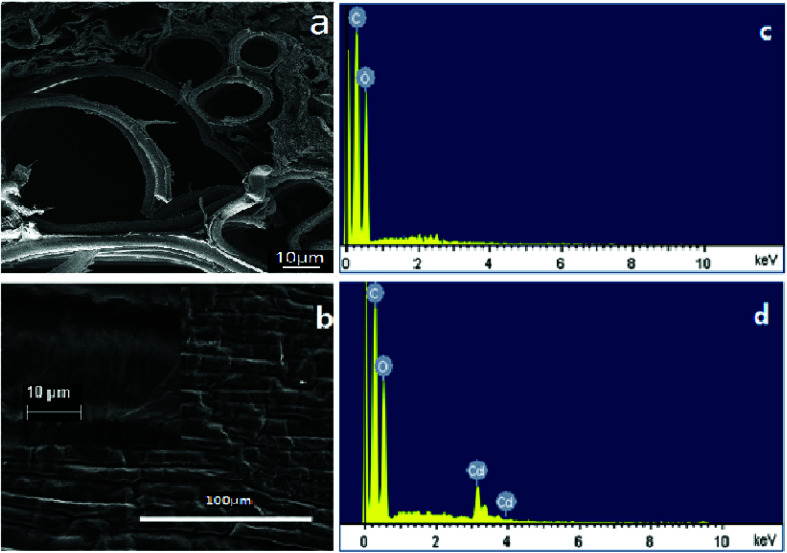
SEM observation of the transversal and flat surface of a piece of garlic peel, and EDS results of garlic peel before and after adsorption of cadmium at pH 4.0.

Fig. 1 presents the SEM observation and EDS results, showing that the transversal surface of a piece of garlic peel has a complicated porous structure, appearing as a main channel with a size of 60 μm, clustered with many smaller channels alongside, constructing the inner structure of garlic peel (as illustrated in Fig. 1a). To our knowledge, there are few works of research about the inner microstructures of the garlic peel, so the porous structure of the garlic peel presented in this study is the first disclosure. While its flat surface is tough and compact, bushy fine hairs grow on the surface when observed at higher resolutions (Fig. 1b). Potentially, this complicated porous and hierarchical structure is favorable for adsorption, and the floating garlic peel comes from particles with large numbers of microsized pores.

EDS results suggest that the main component of the garlic peel is a hydrocarbon compound with some functional groups that are responsible for the capture of cadmium ions from the aqueous solution. After contact with the cadmium solution, the cadmium species appeared in the garlic peel, indicating that the cadmium is adsorbed effectively.

### 3.2 FTIR

**Fig. 2:**
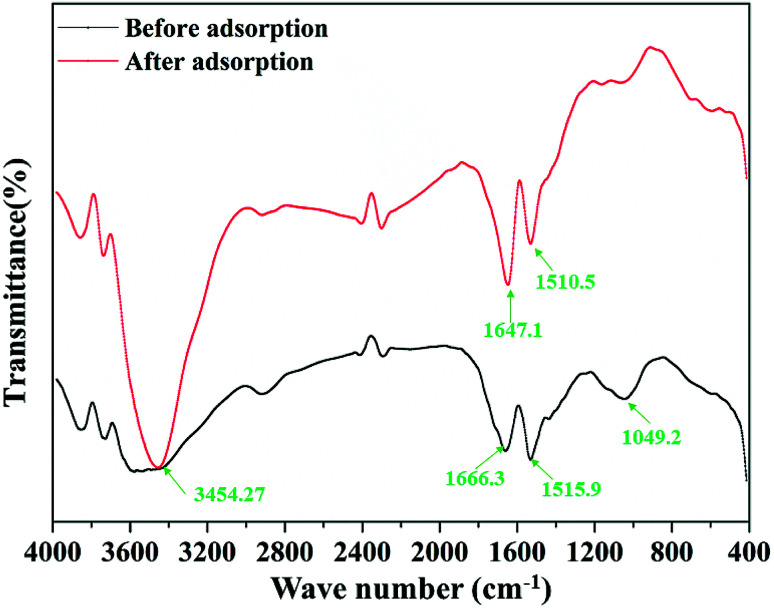
FTIR spectra of the garlic peel before and after adsorption of cadmium at pH 4.0.

Fig. 2 shows the FTIR patterns of the garlic peel before and after contact with the cadmium solution at pH 4.0. In the range of the fingerprint zone, 1500 cm^−1^ to 1700 cm^−1^, the two peaks become drastically stronger, ascribed to the ligand of –COOH, suggesting that the affinity of the cadmium ions to the –COO^−^ occurred through chemical bonding during the adsorption process instead of solely through van der Waals forces. The carboxyl acid functional group is contained in many biomasses, which means that any biomass materials in nature have the potential of being used as the raw materials to prepare the biosorbents. For different biomasses, the content level of such ligands may be quite different and must be comprehensively examined in the selection process. Garlic peel is abundantly planted in many countries and areas, so garlic peel is readily available as an adsorbent source. In most locations, garlic peel is usually considered biowaste with little value, while the present study indicates that it has potential application in water treatment as a low-cost, natural and safe eco-material, that further systematical studying and development would be worthwhile.

### 3.3 Effect of initial concentration of cadmium

**Fig. 3:**
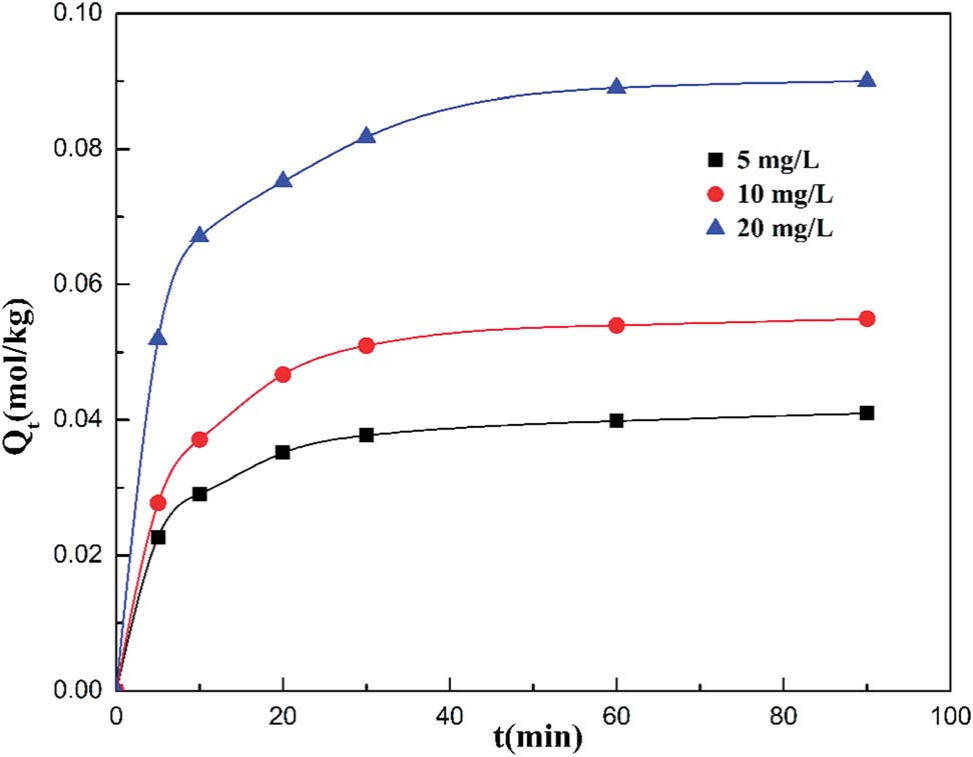
Effect of contact time on adsorption capacity at different initial concentrations of cadmium ion (weight of adsorbent = 300 mg; volume of Cd(ii) solution = 300 mL; equilibrium pH = 4.0; adsorption temperature = 288 K; initial Cd(ii) concentration = 5, 10, 20 mg L^−1^, respectively; citrate concentration = 0.01 mol L^−1^).

Fig. 3 presents the adsorption amount of cadmium on the garlic peel at different initial cadmium concentrations. A higher initial concentration will favor more adsorption amounts of cadmium on the garlic peel. This is common in many biosorption processes due to the larger concentration gradient between the bulk solution and the biosorbent surface. Based on the calculation, it was found that the adsorption process could be described both by the pseudo-first-order kinetic and pseudo-second-order kinetic equations, as shown in the Fig. 1 and 2 of the ESI,† while further analysis shows that the pseudo-second-order kinetic equation is a better fit by comparing with the parameters of pseudo-first-order equation as listed in Table 1, in which the calculation data were more identical with the experimental results. The adsorption constants of *k*_1_ decrease with the higher initial concentration of cadmium, meaning that higher concentrations will slow the adsorption process to reach the equilibrium due to its smaller adsorption kinetic parameters.^33^

**Table 1:** Pseudo-first-order and pseudo-second-order adsorption kinetic constants of Cd(ii) onto the FSGP adsorbent

*Q*_e,exp_ (mg g^−1^)	Pseudo-first-order model			Pseudo-second-order model		
	*k*_1_ (min^−1^)	*Q*_e,cal_ (mg g^−1^)	*R* ^2^	*K*_2_ (10^−2^ g mg^−1^ min^−1^)	*Q*_e,cal_ (mg g^−1^)	*R* ^2^
4.686	0.053	2.367	0.988	0.041	4.926	0.999
6.480	0.050	3.720	0.984	0.023	6.944	0.999
10.651	0.045	6.103	0.986	0.017	11.236	0.998

### 3.4 Effect of temperature

**Fig. 4:**
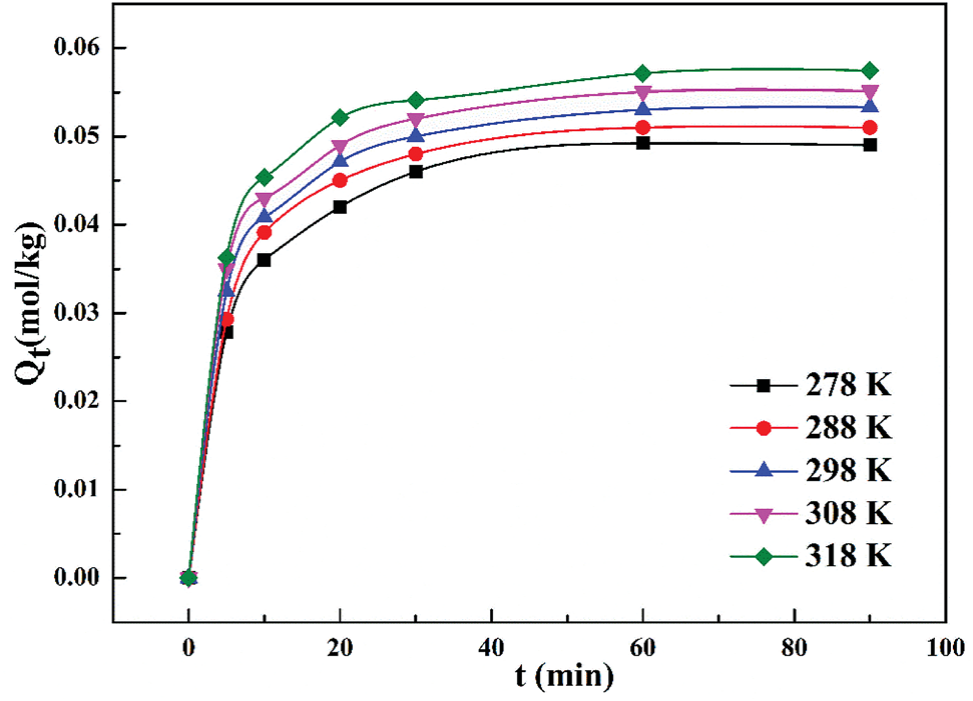
Effect of temperature on the adsorption amount of Cd(ii) on garlic peel (weight of adsorbent = 300 mg; volume of Cd(ii) solution = 300 mL; equilibrium pH = 4.0; adsorption temperature = 278, 288, 298, 308 and 318 K, respectively; citrate concentration = 0.01 mol L^−1^).

**Fig. 5:**
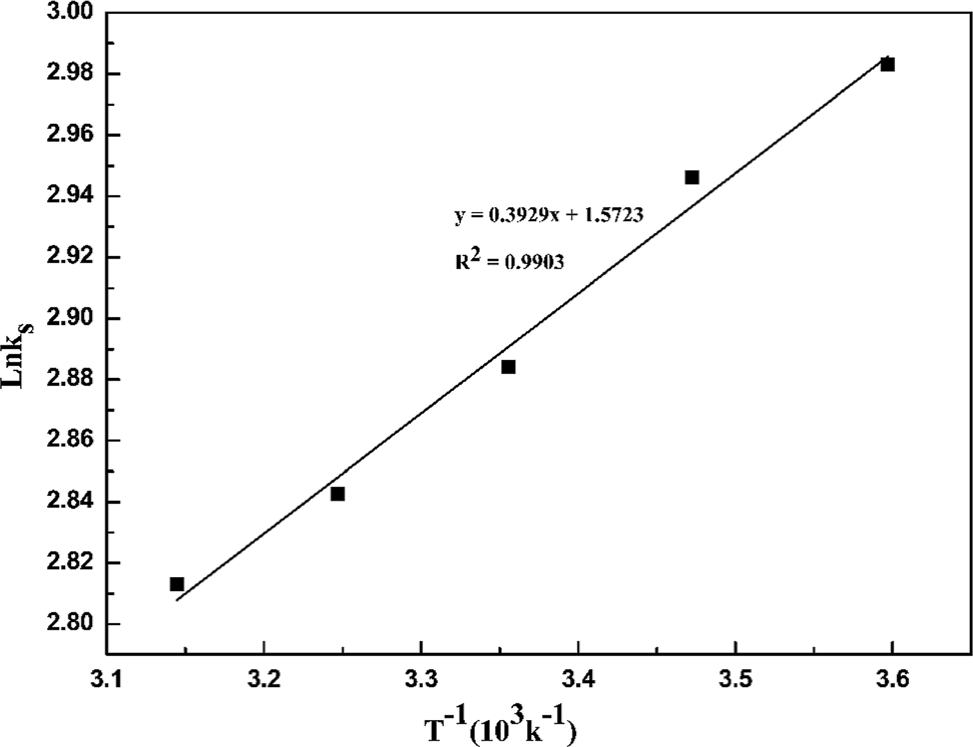
Plot of ln *K*_s_*versus* T^−1^ for cadmium adsorption on saponified garlic peel.

Fig. 4 demonstrates that the adsorption amount of cadmium on garlic peel increases gradually as the temperature increases from 278 K to 318 K. Higher temperature improves the diffusion rate of the cadmium ions because the adsorption process is an endothermic reaction. The pseudo-second-order kinetic equation was found to fit the experimental data very well (as demonstrated in Fig. 3 in ESI†), and further calculation results based on the Arrhenius equation are depicted in Fig. 5. The apparent activation energy of the adsorption was estimated at 3.26 kJ mol^−1^, suggesting that diffusion is the main bottleneck limiting the entire adsorption process. These results also extensively occurred in other cation biosorption processes.^31–33^ Theoretically, effective methods for promoting adsorption should involve decreasing the garlic peel particle size for shorter distance of diffusion, more intensive stirring to thin the liquid layer on the surface of garlic peel, *etc.* Based on above experimental data, the basic thermodynamic parameters including Δ*H*, Δ*S* and Δ*G* were calculated (as demonstrated in Fig. 4 and Table 1 in ESI†), and the Δ*G* values were −0.026 kJ mol^−1^, −0.199 kJ mol^−1^, −0.371 kJ mol^−1^, and −0.543 kJ mol^−1^ for the temperature at 288 K, 298 K, 308 K and 318 K respectively. Therefore, it can be seen that the adsorption process is a spontaneous reaction due to the negative values of Δ*G*.

### 3.5 Effect of dosage

**Fig. 6:**
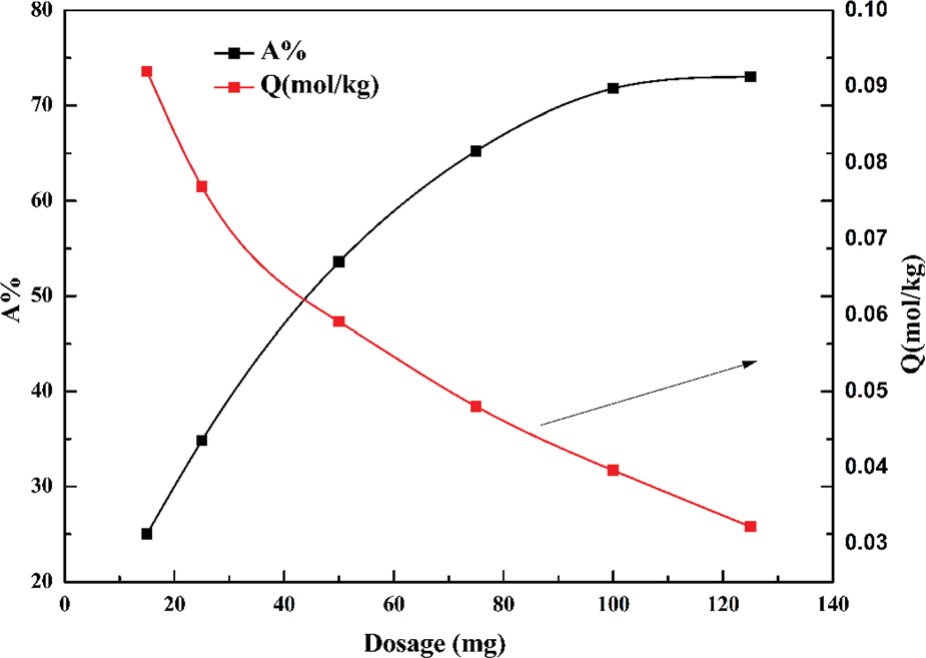
Effect of dosage of garlic peel on the uptake of Cd(ii) (initial concentration of Cd(ii) solution = 10 mg L^−1^; weight adsorbent = 15, 25, 50, 75, 100 and 125 mg, respectively; volume of Cd(ii) solution = 50 mL; initial concentration of citrate = 0.01 mol L^−1^; equilibrium pH = 4.0; contact time = 60 min; temperature = 288 K).

Fig. 6 shows the effect of dosage of garlic peel on the adsorption ratio of cadmium. The removal percentage of cadmium increases with increased garlic peel dosage, and reaches a maximum of 73% at a liquid/solid ratio of 2.0 g L^−1^. The adsorption capacity of cadmium will decrease sharply with larger dosage, which is due to more garlic peel involvement in the adsorption of a certain amount of cadmium leading to the decrease in the utilization efficiency of the functional groups on the garlic peel.^32^

### 3.6 Effect of pH

**Fig. 7:**
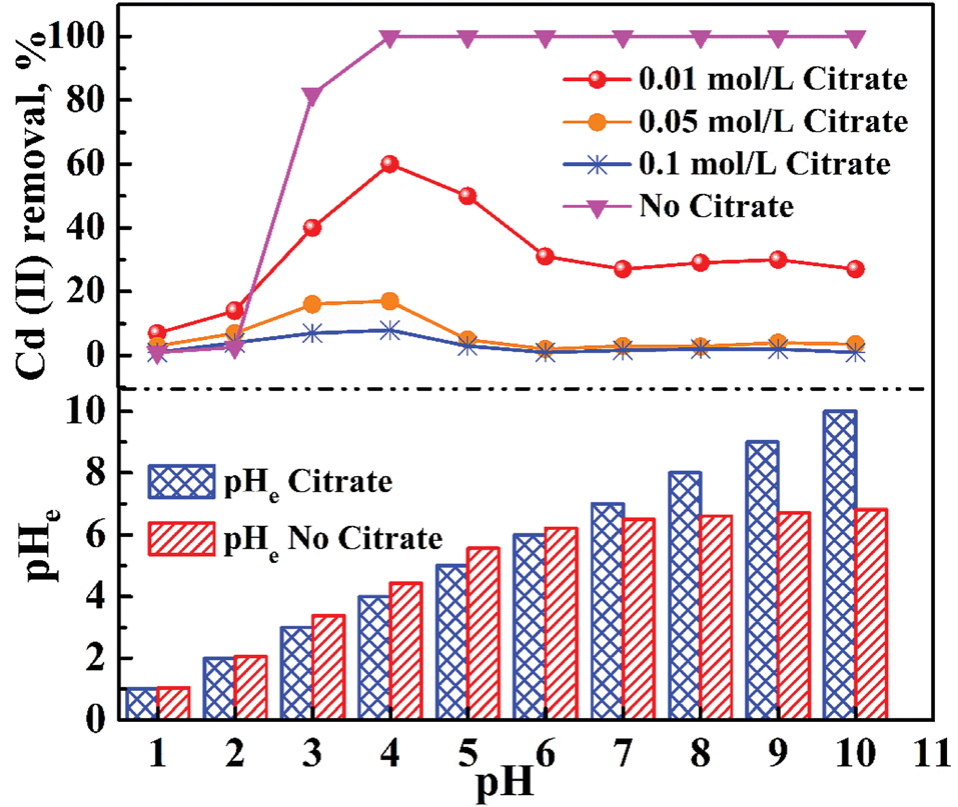
Effect of different equilibrium pH on the adsorption efficiency of cadmium on garlic peel in the presence of citrate. Volume of solution = 50 mL; initial concentration of Cd(ii) = 10 mg L^−1^; weight of garlic peel = 50 mg; contact time = 60 min; temperature = 288 K.

**Fig. 8:**
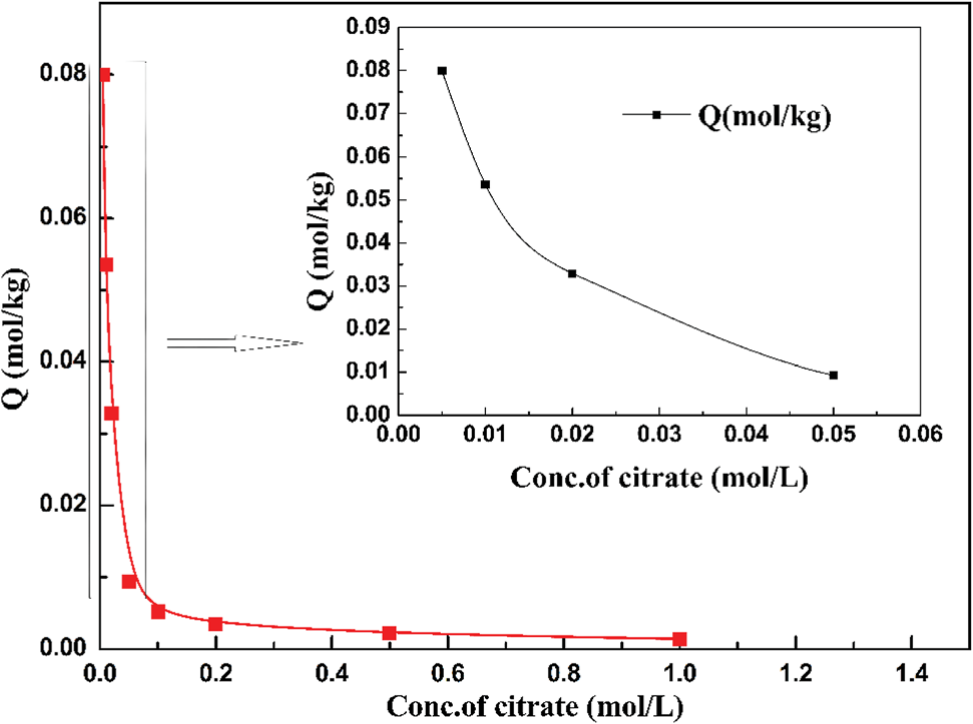
Effect of sodium citrate concentration on Cd(ii) adsorption on garlic peel (initial concentration of Cd(ii) = 10 mg L^−1^; weight of adsorbent = 50 mg; volume of Cd(ii) solution = 50 mL; equilibrium pH = 4.0; adsorption temperature = 288 K).

Fig. 7 shows the adsorption behavior of cadmium on the garlic peel. At a pH near 4.0, cadmium reaches its maximum adsorption, while in the absence of citrate, the maximum adsorption capacity value will keep to a constant, and similar phenomena were reported in other adsorption systems.^34^ And it is also found that the presence of citrate has the strong buffer capability to keep the pH constant during the adsorption process. Obviously, the presence of citrate causes this particular adsorption behavior. Additional citrate in the solution will more drastically inhibit the cadmium adsorption on the garlic peel. A cadmium-bearing solution with different concentrations of citrate was prepared to test the adsorption behavior of garlic peel, and as shown in Fig. 8, even very small amount of citrate will cause a sharp decrease of cadmium adsorption efficiency on garlic peel.

**Fig. 9:**
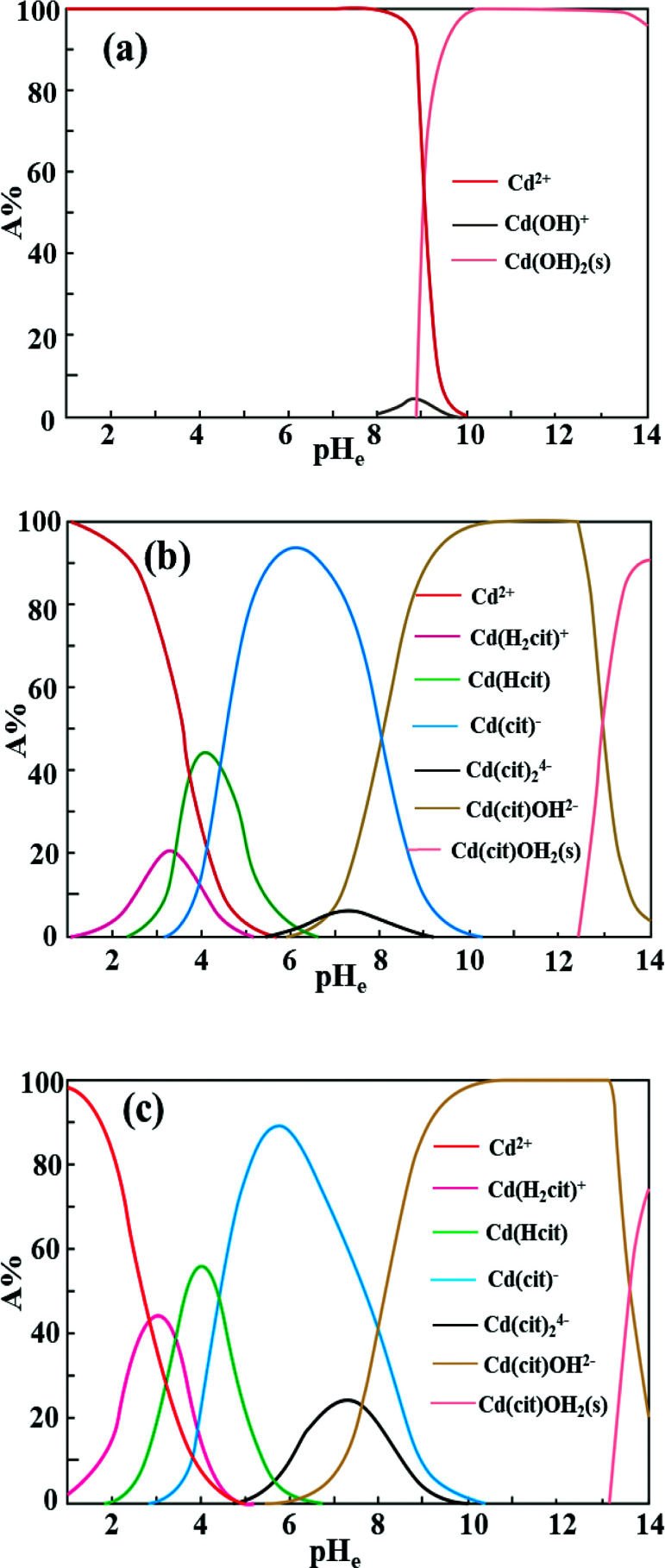
Species distribution of cadmium and citrate in the solution Cd(ii)–C_3_H_5_O(COOH)_3_–H_2_O (0.1 mmol L^−1^ Cd(ii) in the solution, and initial citric acid concentration is 0, 0.01, 0.05 mol L^−1^, respectively).

Citrate is a strong chelating reagent that can form complex compounds with cadmium in the form of various coordination species, which may show different affinity to the functional groups in the garlic peel biosorbent. In order to learn more about the adsorption process, the cadmium species in the citrate solution are necessary to be demonstrated. Based on the mass balance and thermodynamic equilibrium theories,^35^ the species distributions of cadmium in the presence of citrate are calculated and depicted in Fig. 9, the dominant cadmium species is Cd^2+^ at pH < 2.0; and then a series of complex of cadmium and citrate appears in the species of Cd(H_2_cit)^+^, Cd(Hcit)^0^, and Cd(cit)^−^ with the pH increasing, which have less positive charge and even negative charge that will delay or prevent their approaching to the –COO^−^ ligands of garlic peel biosorbent. And this is quite identical to the adsorption results demonstrated in Fig. 7 and 8, suggesting that the appearance of the species of Cd(H_2_cit)^+^, Cd(Hcit)^0^, and Cd(cit)^−^ is the main factor causing the decrease of adsorption capability when citrate concentration increases. At much higher pH above 5, the species of Cd(cit)^−^ is dominant that will be strongly repelled by the negative charge of –COO^−^ of garlic peel, let alone further adsorbed on the garlic peel by chemical binding.

### 3.7 Effect of coexisting ions

**Fig. 10:**
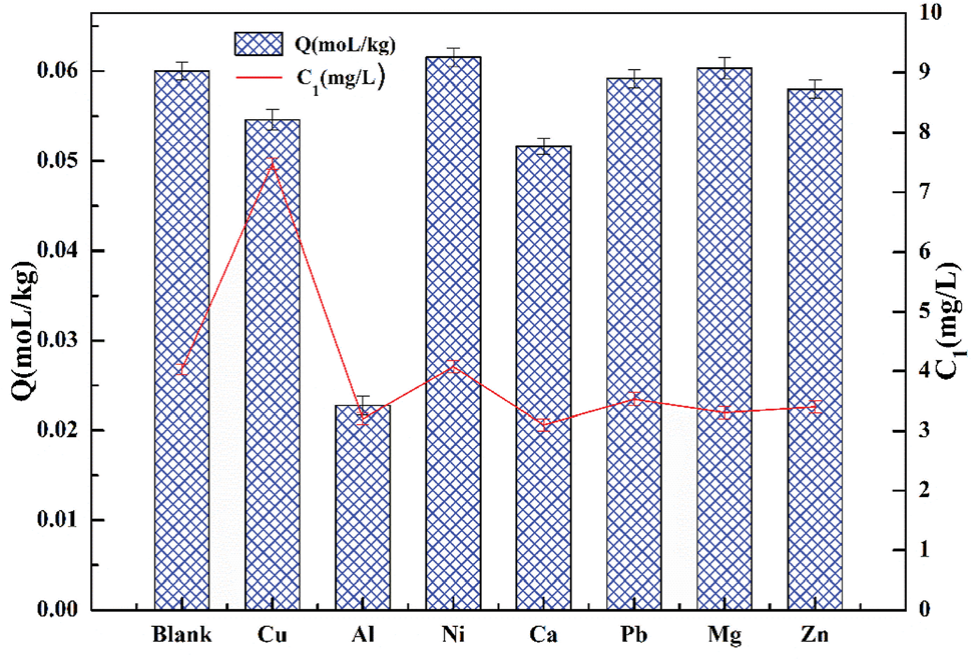
Effect of coexisting ions on the cadmium adsorption on saponified garlic peel (initial concentration of Cd(ii) = 10 mg L^−1^; citrate concentration = 0.01 mol L^−1^; weight of adsorbent = 50 mg; volume of Cd(ii) = 50 mL; equilibrium pH = 4.0; coexisting ions = Mg(ii), Zn(ii), Cu(ii), Ca(ii), Al(iii), Pb(ii), Ni(ii), with an initial concentration for each metal ion of 10 mg L^−1^).

Fig. 10 demonstrates the effect of competitive ions on the adsorption of cadmium on garlic peel. Ni^2+^, Pb^2+^ and Mg^2+^ ions appear to have no effect on the adsorption of cadmium, while the other ions do have an effect according to the following order: Al^3+^ > Ca^2+^ > Cu^2+^ > Zn^2+^. The competitive adsorption for cadmium from other coexisting ions is also quite different from that in the absence of citrate. In our previous research, Pb^2+^ and Cu^2+^ both would be adsorbed preferentially over Cd^2+^ in the absence of citrate, while in the present study, their competitive adsorption over cadmium does not occur, which means that citrate has a stronger chelating affinity to Pb^2+^ and Cu^2+^ ions to form coordination complexes than that of the Cd^2+^ ion.

### 3.8 Adsorption of cadmium from leach liquor of real soil

To check the removal efficiency of cadmium on the floatable garlic peel, a soil sample was taken from the neighbor area of a smelter in Zhuzhou city, and 50 g wet sample (60% water content) was placed in a beaker with 200 mL water containing 0.01 mol L^−1^ citric acid, kept stirring for 2 h. And then 150 mL leach liquor with pH 4.3 was added with 450 mg dried floatable saponified garlic peel, shaking for 4 h at a reciprocating rate of 110 rpm. The cadmium concentration in the leach liquor before and after adsorption was 15.8 mg L^−1^ and 4.9 mg L^−1^ respectively, suggesting 69.0% cadmium in the solution had been adsorbed. More twice adsorption was conducted, and the final concentration of cadmium in the solution was only 0.11 mg L^−1^, which indicated that the cadmium in the real leach liquor could be removed almost completely by multiple-stage adsorption process. And such adsorption capability could be kept almost the same level even after 10 times adsorption/adsorption operations by checking with the real leach liquor of Cd-contaminated soil with 0.01 mol L^−1^ citric acid. Based on above results, a principal flowsheet of soil washing coupled with cadmium recovery *via* biosorption was proposed for the cadmium-contaminated soil remediation (as demonstrated in Fig. 7 in ESI†).

### 3.9 Adsorption isotherm

**Fig. 11:**
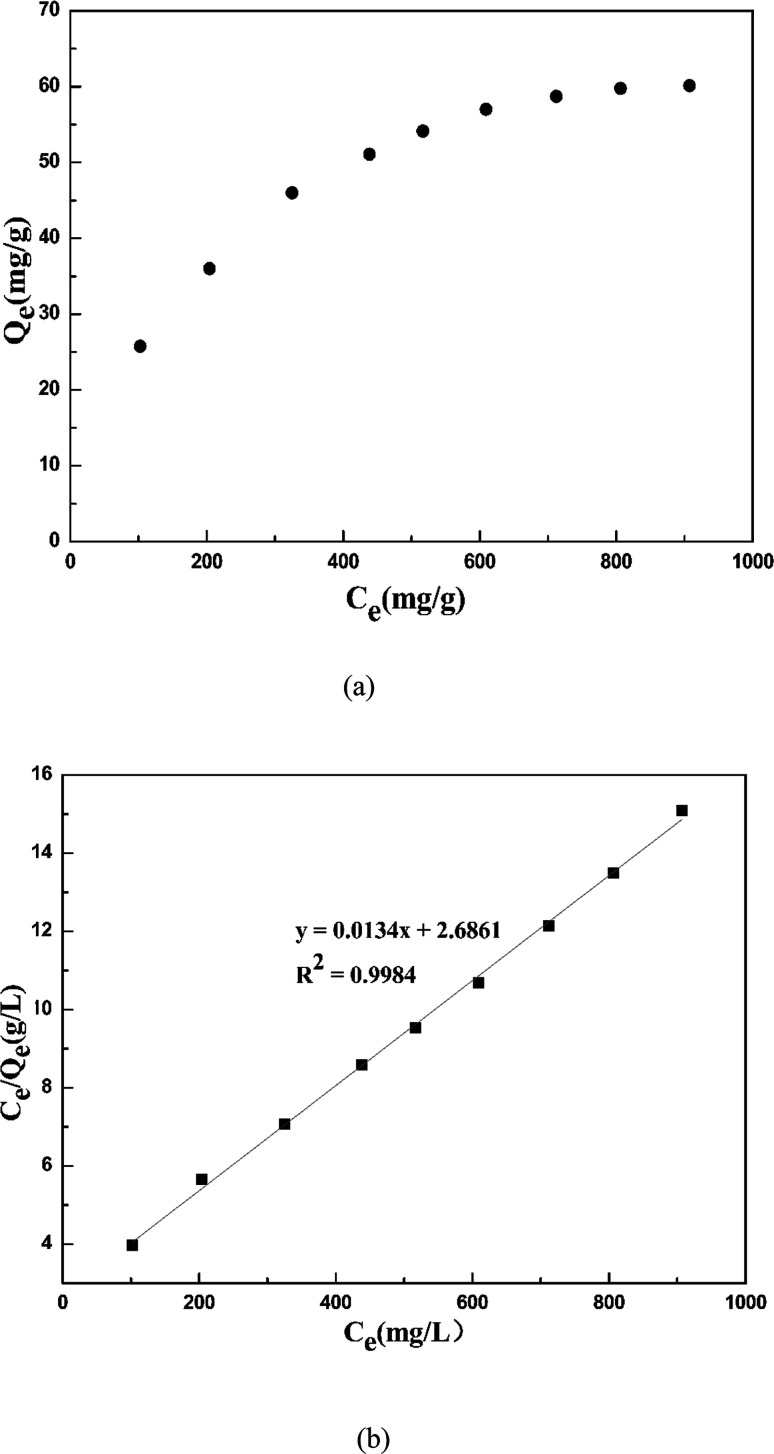
(a) Plot of *Q*_*t*_ ∼ *C*_e_ at 288 K (weight of adsorbent = 50 mg; volume of Cd(ii) solution = 50 mL; equilibrium pH = 4.0; contact time = 60 min, sodium citrate concentration = 0.01 mol L^−1^); and (b) Langmuir plot at 288 K.

Fig. 11 shows the isotherm adsorption behavior of cadmium ions on garlic peel in the presence of 0.01 M citrate. Based on the experimental data (upper figure in Fig. 11), the maximum adsorption capacity of cadmium ions on garlic peel is 60.11 mg g^−1^, or 0.53 mol-Cd/kg-FSGP, which is comparable with other biosorbents and ion exchange resins.^36^ Additionally, the isotherm data fit the Langmuir model well (bottom figure in Fig. 11), and the theoretical calculation value of the maximum capacity of cadmium ions on garlic peel was evaluated as 74.63 mg g^−1^ (0.66 mol-Cd/kg-FSGP). The adsorption constant of *K*_L_ is 0.005 L mg^−1^, which is much less than 1.0, indicating that the adsorption of cadmium ions on garlic peel is a spontaneous process (Table 2).^37–40^

**Table 2:** Langmuir adsorption isotherm parameters of the FSGP adsorbent

Langmuir isotherm model			
*Q*_e,exp_ (mg g^−1^)	*Q*_e,cal_ (mg g^−1^)	*K*_L_ (L mg^−1^)	*R* ^2^
60.11	74.626	0.005	0.998

For comparison, the maximum adsorption capacity of various adsorbents reported in the literature are listed in Table 3; garlic peel shows good adsorption capacity even when compared with common adsorbents.

**Table 3:** Maximum adsorption capacity of various adsorbents for cadmium removal

Adsorbents	Adsorption pH and other conditions	Maximum adsorption capacity, mg g^−1^	Reference
Fe_3_O_4_@APS@AA-*co*-CA MNPs	5.5	29.6	41
Manganoxide mineral	6.0	6.8	42
Turkish illitic clay	4.0	13.09	43
Red mud	6.5	17.98	44
Kaolinite	5.5	14.39	45
Colemanite ore waste	5.0	29.7	46
Flue gas desulphurization gypsum	7.0	32.57	47
Chrysotile	5.5	23.82	48
GMZ bentonite	6.5	11.00	49
Activated carbon	5.0	33.57	50
Floatable saponified garlic peel	4.0 with 0.01 M citrate	60.11	This study

### 3.10 Repeatable adsorption of GP

In order to check the repeatable use of GP for adsorption, the saponified garlic peel was used to perform 10 cycles of adsorption and elution (as shown in Fig. 5 in ESI†). No obvious decrease of adsorption capability was observed, meaning that the saponified garlic peel is durable enough in mechanical strength and retained its functional sites during the adsorption/elution operations. Unlike most of the common biosorbents that are easily breakable when soaked in water, the cellulose and hemicellulose content of the saponified garlic peel is as high as 76% of its mass,^51^ making it strong and tough, and its active level of acidic functional groups is determined by titration as rich as 12% of its mass for effective adsorption. The above merits in both the mechanical strength and chemical binding ligands makes the garlic peel an excellent biosorbent.

### 3.11 Mechanism of modification and adsorption

Based on the above results, the saponification and cadmium adsorption mechanisms were deduced to be performed according to a cation exchange process, in which the cations or molecules in the aqueous solution will replace the Na^+^ or Ca^2+^ bonded with –COO^−^ ligands in the garlic peel. The possible mechanism during the saponification and adsorption processes, could be listed as following: GP-COOH + NaOH = GP-COONa + H_2_O (saponification), and 2GP-COONa + Cd^2+^ = (2GP-COO)–Cd + 2Na^+^ (adsorption), respectively. The saponification process of the garlic peel modification belongs to a type of neutralization of acidic substances contained in the garlic peel through contact with the alkaline solution. Hence, the adsorption of cadmium ions on the modified garlic peel can be understood by a cation exchange process. While for the solution in the presence of citrate from pH 2 to 10, cadmium will existed in the form of Cd(H_2_cit)^+^, Cd(Hcit)^0^, and Cd(cit)^−^ species that will delay or prevent the adsorption due to the weaker attraction or repelling under the electrostatic interaction between the cadmium species and –COO^−^ ligands on garlic peel.

In order to learn more about the adsorption mechanism, an X-ray photoelectron spectroscopy was used for the determination of the binding sites of cadmium on the modified garlic peel. That is, 300 mg of ground FSGP particles was exposed to 300 mL of 20 mg L^−1^ cadmium solution (0.01 mol L^−1^ citrate) at pH 4.0 for 1 h, and the FSGP particles were filtered and dried in an oven at 80 °C, then the dried sample was analyzed by XPS (as shown in Fig. 6 of ESI†), showed the XPS of Cd 3d spectrum on the FSGP particles. The most intense Cd peaks were observed at 405.1 eV, suggesting that cadmium was adsorbed on the FSGP particles through surface complex with carboxylic groups –COOH or the deprotonated form −COO^−^. Additionally, the symmetrical peak at 405.1 eV indicates that cadmium ion adsorbed on the surface of the FSGP particles existed only in a single oxidation state of divalent cadmium. Obviously, it's easy to understand that the adsorption of cadmium on the garlic peel is a cation exchange process, without causing the valent transformation of the cadmium.

## 4. Conclusion

The porous structures of the garlic peel were disclosed for the first time by SEM observation, proving garlic peel's low density and excellent adsorption capacity for metal ions, such as cadmium, in the present study. The garlic peel with closed pores can float on the solution surface, which facilitates collection with a squeegee after adsorption. The presence of citrate in the aqueous solution causes a drastic adsorption decrease of cadmium onto the micronized garlic peel due to the formation of complex molecules coordinated between cadmium ions and citrate, which is not easily adsorbed probably due to the weaker attraction or repelling under the electrostatic interaction between the cadmium species and –COO^−^ on garlic peel. The maximum of adsorption capacity at pH 4.0 in the presence of 0.01 mol L^−1^ citrate is 60.11 mg-Cd/g-FSGP, which is comparable to most adsorbents. The apparent activation energy of the adsorption process is 3.26 kJ mol^−1^, suggesting the diffusion step limited the whole adsorption process. Generally, above results can serve as a good reference for cadmium-contaminated soil remediation by coupling citric acid washing with subsequent biosorption treatment. This novel procedure has great potential of being superior to other remediation technique systems because it is more economical, ecofriendly and simple to handle. Citric acid is biodegradable, and its secondary hazardous damage to the environment can be negligible. Meanwhile, the remaining citric acid in the washing water can be reused to treat the soil in the next cycles, and the heavy metal ions were selectively recovered by garlic peel adsorption.

## Conflicts of interest

There are no conflicts to declare.

## Supplementary Material

RA-008-C8RA03502D-s001
