# Supplementary material for: Removal of cadmium from a citrate-bearing solution by floatable microsized garlic peel
Source: RSC Adv. 2018 Aug 7;8(50):28284–92. doi: 10.1039/c8ra03502d (PMC9084252; doi:10.1039/c8ra03502d)
Supplement: RA-008-C8RA03502D-s001 [file RA-008-C8RA03502D-s001.pdf]

Electronic Supplementary Material (ESI) for RSC Advances.
This journal is © The Royal Society of Chemistry 2018

Supporting Information for

Removal of cadmium from citrate-bearing solution by floatable microsized garlic peel

Jiangang Sun[a, #], Lipu Yin[a], Xiaohui Li[b, *], Xianbin Ai[a], Ying Huang[c], YanliYin[c], Junyou Liu[c], Kai Huang [a,*]

[a]School of Metallurgical and Ecological Engineering, University of Science and Technology Beijing, Xueyuan Rd.30, Haidian District, 100083, Beijing city, China

[b]Institute of Bioresource, Jiang Xi province, Changdong Rd. 7777, Nanchang City, China

[c]Beijing Keda Advanced Technology Company, Xueyuan Rd.30, Haidian District, 100083, Beijing city, China

AUTHOR INFORMATION:

Corresponding Author

*E-mail: khuang@metall.ustb.edu.cn. Tel.: +86-10-62334204.

*E-mail: lixiaohui211121@163.com; Tel.: +86-10-82375042.

Fig. 1 Pseudo-first-order kinetics plots for adsorption of Cd(II) onto FSGP at 288 K at different initial concentrations of cadmium ions (same adsorption conditions as stated in Fig. 3 of the context)

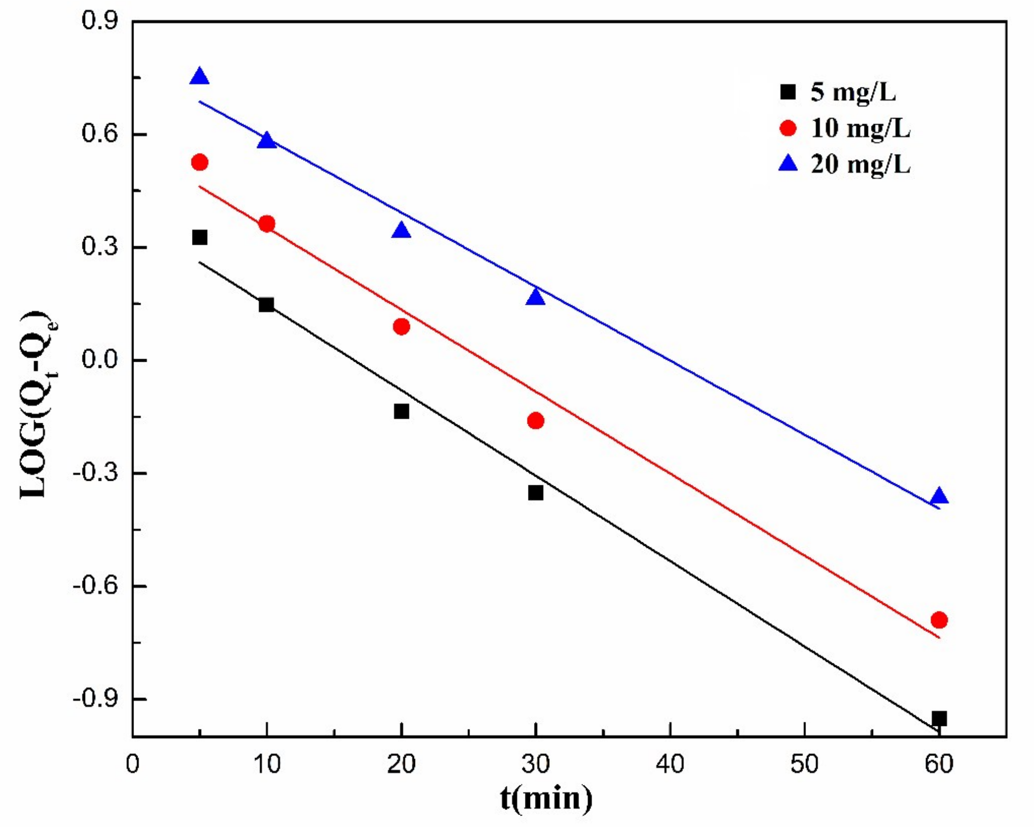

t (min)	Log (Q_e-Q_t)		
	5mg/L	10mg/L	20mg/L
5	0.33	0.53	0.75
10	0.15	0.36	0.58
20	-0.14	0.09	0.34
30	-0.35	-0.16	0.16
60	-0.95	-0.69	-0.36

Fig. 2 Pseudo-second-order kinetic plots for adsorption of Cd(II) onto FSGP at 288 K

at different initial concentrations of cadmium ions (same adsorption conditions as stated in Fig. 3 of the context)

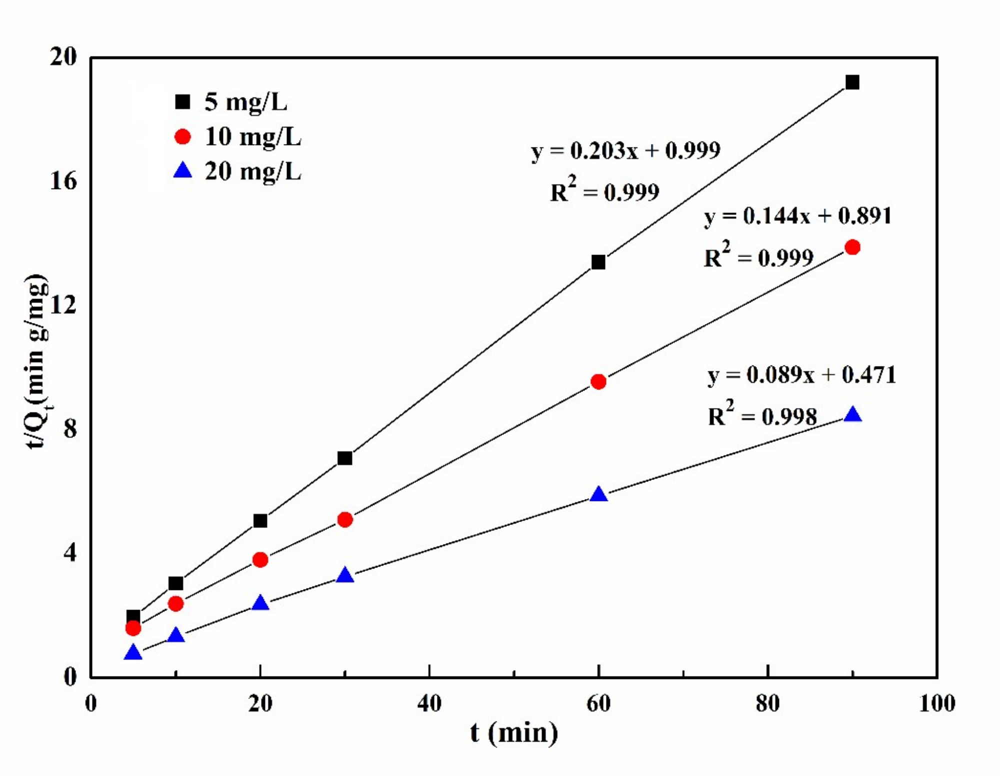

t (min)	t/Q_t (min g/mg)		
	5mg/L	10mg/L	20mg/L
5	1.96	1.60	0.78
10	3.05	2.39	1.33
20	5.06	3.81	2.36
30	7.08	5.10	3.26
60	13.41	10.04	5.87
90	19.21	13.89	8.45

Fig. 3 Pseudo-second-order plots at different temperature (same adsorption

conditions as stated in Fig. 4 of the context)

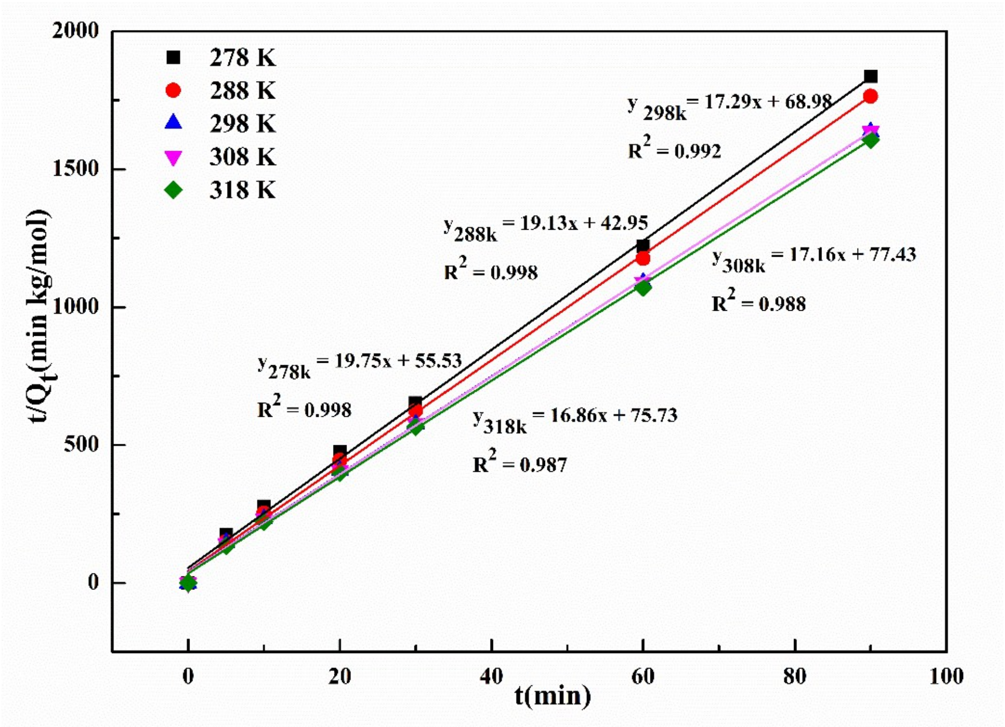

t (min)	t/Q_t (min kg/mol)				
	278 K	288 K	298 K	308 K	318 K
5	173.5	150.1	147.1	142.9	134.9
10	277.8	253.2	232.6	232.6	222.5
20	476.2	444.5	408.2	408.2	400.1
30	652.2	625.1	576.9	576.9	566.1
60	1219.2	1176.5	1090.3	1090.3	1071.4
90	1836.7	1764.7	1636.3	1636.4	1607.1

Fig.4 Curve obtained by thermodynamic calculation

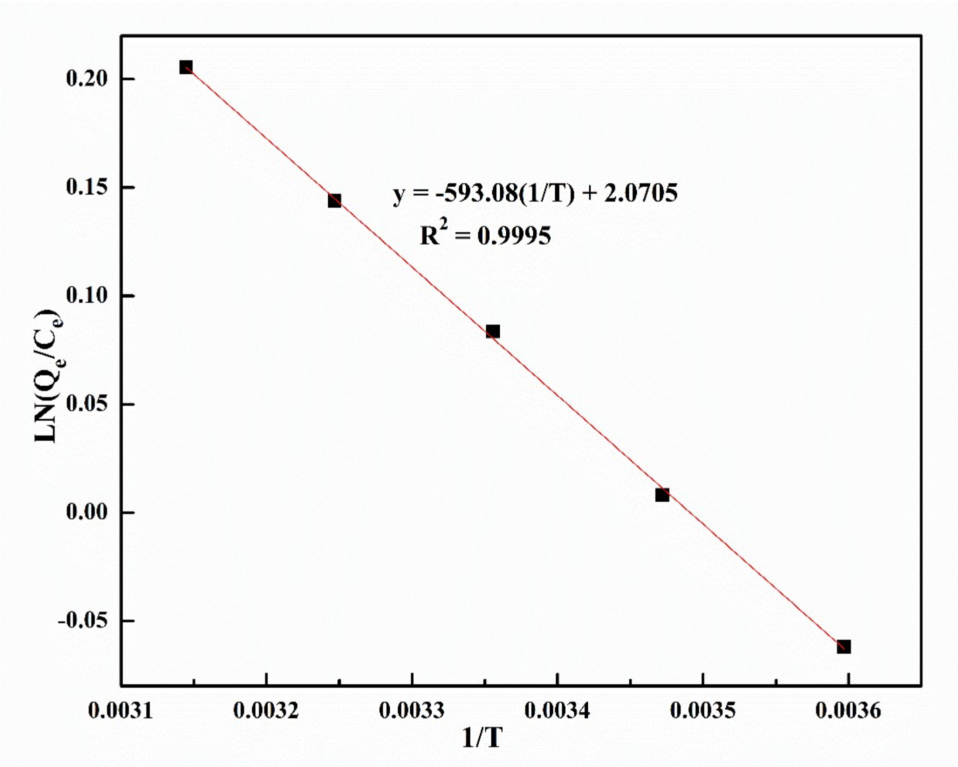

Equations for calculation:

$$\Delta G = -RT\ LnK$$

$$\Delta G = \Delta H - T\Delta S$$

$$LnK = -\frac{\Delta H}{RT} + \frac{\Delta S}{R}$$

$$K = q_e/Ce = \frac{(C_. - Ce)V}{Ce\ m}$$

Table 1

C_0 (mg/L)	ΔH (KJ/mol)	ΔS (J/mol)	ΔG/(KJ/mol)			
			288K	298K	308K	318K
10	4.93	17.21	-0.026	-0.199	-0.371	-0.543

Fig. 5 Effect of adsorption and elution cycles on the cadmium adsorption on FSGP

particles

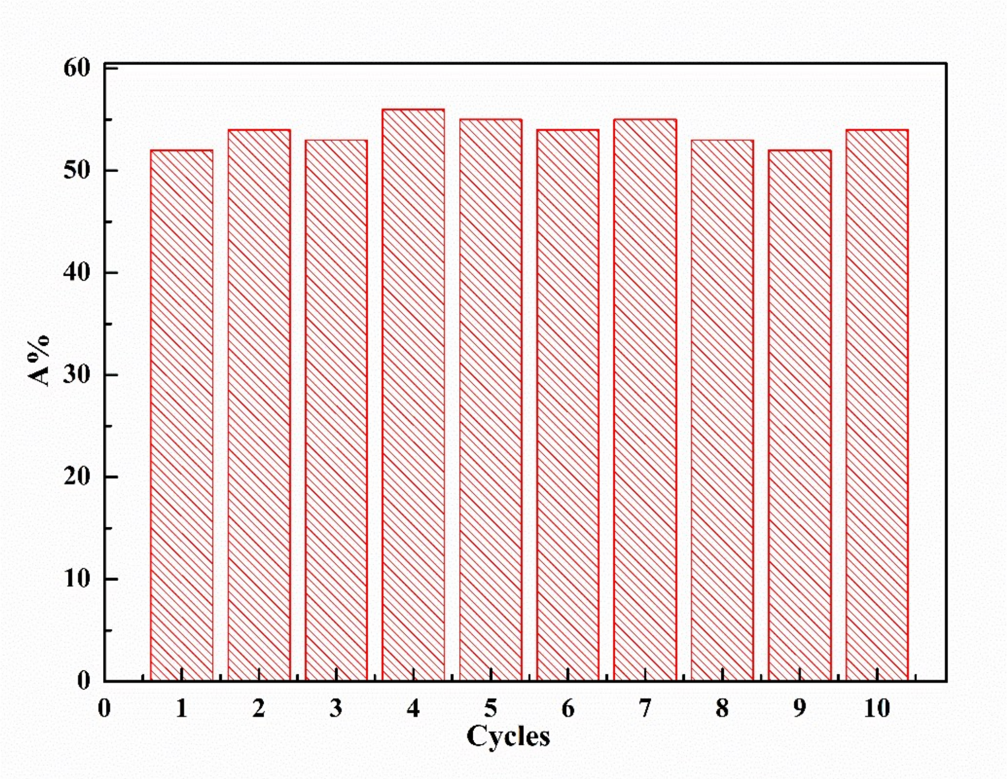

Fig. 6 XPS of the FSGP particles before and after adsorption of Cd(II) (weight of

adsorbent=300 mg; volume of Cd(II) solution=300 ml; equilibrium pH=5.0; adsorption temperature=288K; contact time=60 min, cadmium concentration=20mg/L, citrate concentration=0.01 mol/L)

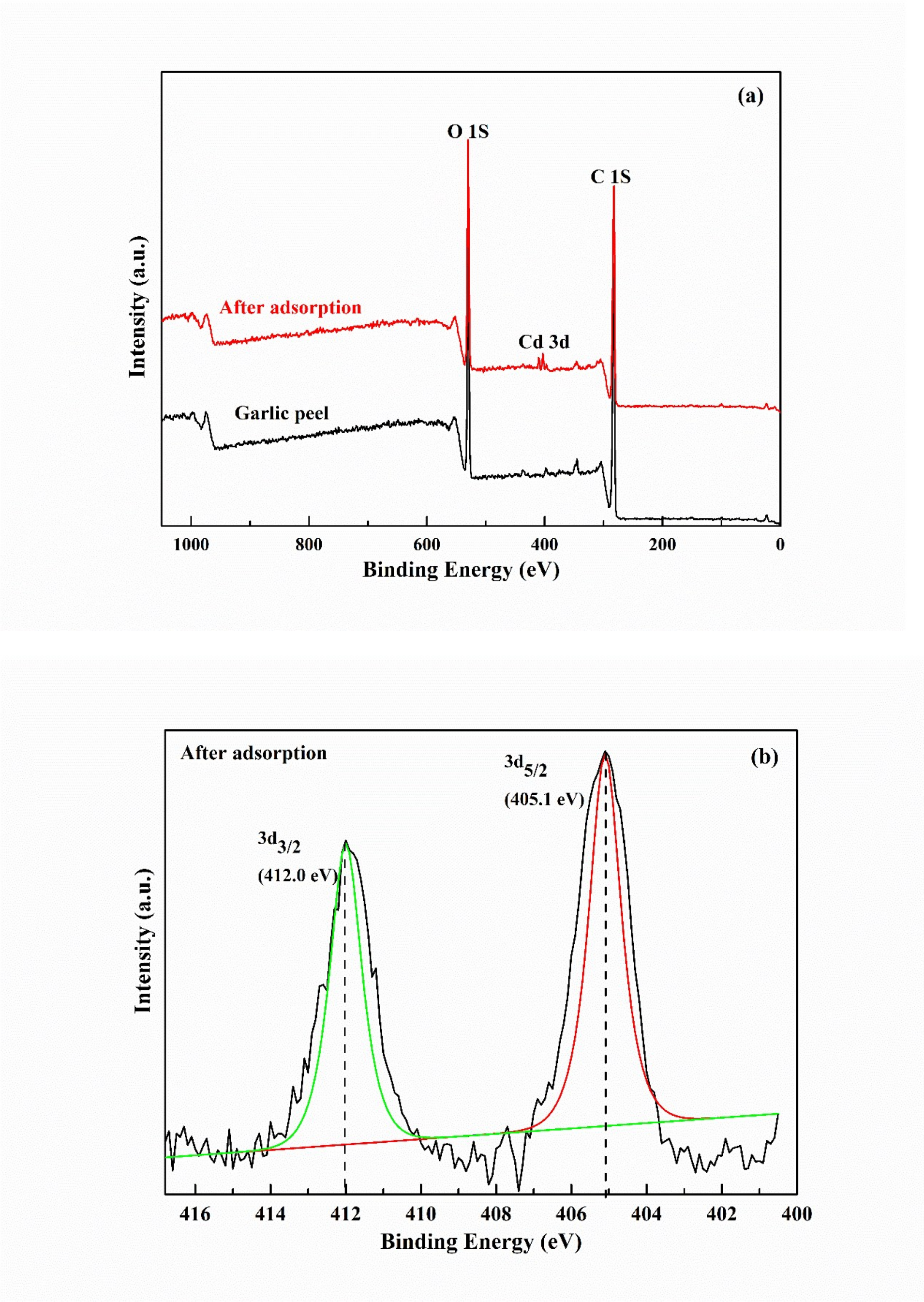

Fig. 7 Flowsheet of contaminated soil remediation by washing and adsorption process

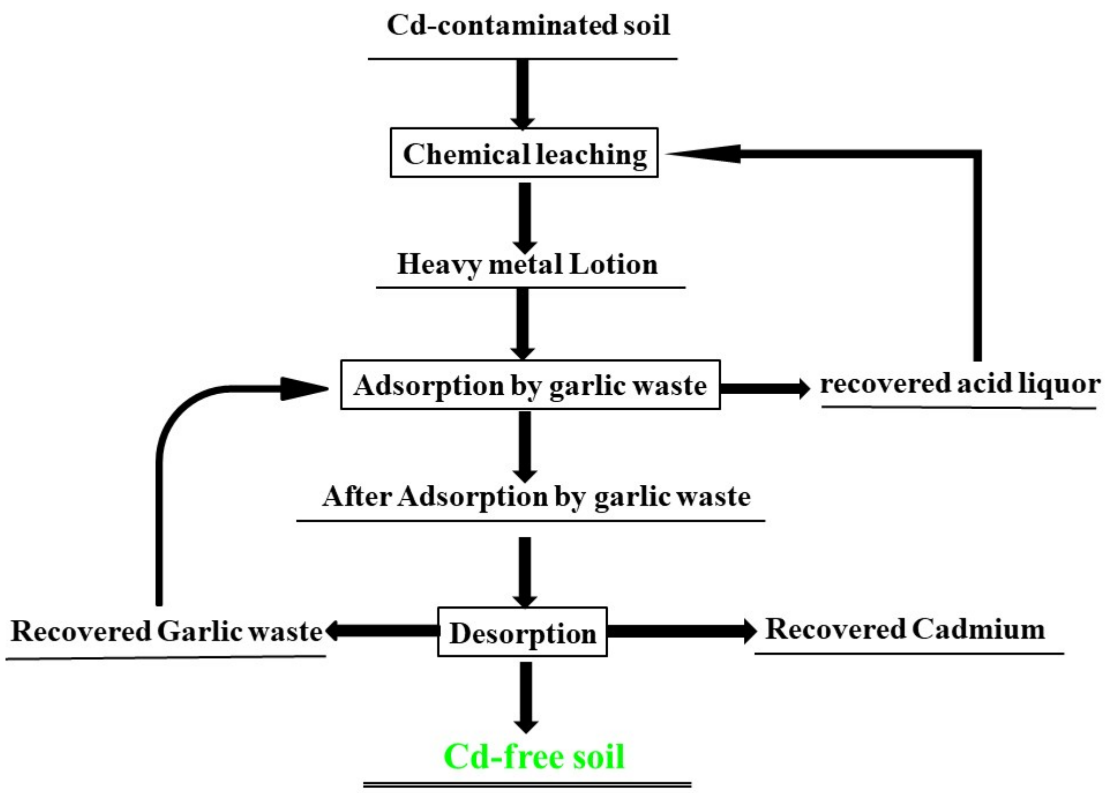
Cd-contaminated soil
Chemical leaching
Heavy metal Lotion
Adsorption by garlic waste
recovered acid liquor
After Adsorption by garlic waste
Recovered Garlic waste
Desorption
Recovered Cadmium
Cd-free soil
